# Th17 Down-regulation Is Involved in Reduced Progression of Schistosomiasis Fibrosis in ICOSL KO Mice

**DOI:** 10.1371/journal.pntd.0003434

**Published:** 2015-01-15

**Authors:** Bo Wang, Song Liang, Yu Wang, Xing-Quan Zhu, Wei Gong, Hui-Qin Zhang, Ying Li, Chao-Ming Xia

**Affiliations:** 1 Department of Parasitology, Medical College of Soochow University, Suzhou,Jiangsu Province, The Peoples Republic of China; 2 State Key Laboratory of Veterinary Etiological Biology, Key Laboratory of Veterinary Parasitology of Gansu Province, Lanzhou Veterinary Research Institute, Chinese Academy of Agricultural Sciences, Gansu Province, The Peoples Republic of China; University of Manchester, UNITED KINGDOM

## Abstract

**Background:**

Granulomatous and fibrosing inflammation in response to parasite eggs is the main pathology that occurs during infection with *Schistosoma* spp. CD4+ T cells play critical roles in both host immune responses against parasitic infection and immunopathology in schistosomiasis,and coordinate many types of immune cells that contribute to fibrosis. ICOSL plays an important role in controlling specific aspects of T cell activation, differentiation, and function. Previous work has suggested that ICOS is essential for Th17 cell development. However, the immunopathogenesis of this pathway in schistosomiasis fibrosisis still unclear.

**Methodology/Principal Findings:**

Using models of schistosomiasis in ICOSL KO and the C57BL/6 WT mice, we studied the role of the ICOSL/ICOS interaction in the mediation of the Th17 response in host granulomatous inflammation, particularly in liver fibrosis during *S. japonicum* infection, and investigated the immune responses and pathology of ICOSL KO mice in these models. The results showed that ICOSL KO mice exhibited improved survival, reduced liver granulomatous inflammation around parasite eggs, markedly inhibited hepatic fibrosis development, lower levels of Th17-related cytokines (IL-17/IL-21), Th2-related cytokines (IL-4/IL-6/IL-10), a pro-fibrotic cytokine (IL-13), and TGF-β1, but higher level of Th1-related cytokine (IFN-γ) compared to wild-type (WT) mice. The reduced progression of fibrogenesis was correlated with the down-regulation of Th17 and Th2 and the elimination of ICOSL/ICOS interactions.

**Conclusions/Significance:**

Our findings suggest that IL-17-producing cells contribute to the hepatic granulomatous inflammation and subsequent fibrosis. Importantly, there was a clearly positive correlation between the presence of IL-17-producing cells and ICOS expression in ICOSL KO mice, and additional results indicated that Th17 was involved in the pathological tissue remodeling in liver fibrosis induced by schistosomiasis.

## Author Summary

The full activation and differentiation of T cells into Th1, Th2 or Th17 cells requires costimulatory molecules and cytokines. ICOS has also been implicated in chronic inflammation and is critical for Th17 cell development. CD4^+^ IL-17-secreting T cells have been shown to contribute to pathology in some models of liver fibrosis. However, neither the significance nor the immunopathogenesis of this pathway have been elucidated in schistosomiasis fibrosis. The present study used the ICOSL KO mice to assess the role of the ICOSL/ICOS interaction in the mediation of the Th17 response in host granulomatous inflammation, particularly in liver fibrosis during *S.japonicum* infection. This study further clarifies the immune regulatory mechanism of fibrosis and sheds light on the understanding of the immunopathogenesis of *Schistosoma*-induced fibrosis. It might reveal new therapeutic targets that interfere with Th17 cell migration or differentiation in granulomas and the subsequent fibrosis following infection with *S. japonicum*.

## Introduction

T cell activity is regulated by a complex network of transmembrane receptor/ligand pairs that act in synchrony with the T cell receptor (TCR) to inhibit (co-inhibition) or enhance (co-stimulation) immunity [1]. ICOS, a member of the CD28 family of co-receptor molecules whose expression is induced on activated T cells, was discovered over a decade ago using antibodies raised against activated human T cells and cloned as a 2.6 kb complementary DNA sequence encoding a protein with 39% similarity to human CD28 [2]. The ligand of ICOS, ICOSL (B7h, GL50, LICOS, B7RP-1), is constitutively expressed at low levels on B cells, macrophages and dendritic cells, and ICOSL expression is further up-regulated upon activation of these cells [3]. The ICOS ligand (ICOSL) plays an important role in controlling specific aspects of T cell activation, differentiation, and function [2,4]. Both ICOS-deficient and ICOSL-deficient mice have defects in humoral immunity characterized by decreased levels of IgE and IgG1 in the serum and defects in antibody class switching and GC formation [4,5].

Fibrosis is a normal consequence of tissue injury and chronic inflammation and is characterized by the accumulation and activation of excessive numbers of fibroblasts, the deposition of extracellular matrix (ECM)proteins such as collagen, and the distortion of normal tissue architecture [6]. Although fibrosis typically begins as part of wound healing, the excessive accumulation of collagen and other ECM components during chronic inflammation can lead to the destruction of normal tissue architecture and the loss of function [6]. Thus, fibrosis is a major cause of morbidity and mortality worldwide [7,8].

Granulomatous and fibrosis inflammation in response to parasite eggs is the main pathology that occurs during infection with *Schistosoma* spp. [9,10]. CD4^+^ helper T cells adapt and amplify their responses to match different categories of infections and coordinate many types of immune cells that contribute to fibrosis [11–13]. Mice infected with *Schistosoma mansoni* develop hepatic granulomas around parasite eggs [14]. However, concurrent immune responses to an extremely diverse repertoire of antigens cause marked exacerbation of the fibrosis in an environment characterized by high levels of IL-13 and IL-4 in the chronic phase of infection [6,15]. Reciprocal APC and CD4^+^ T cell activation following stimulation with egg Ag leads to the increased expression of the co-stimulatory molecules CD80 and CD86, the secretion of proinflammatory cytokines and chemokines that cause increased lesional recruitment of neutrophils, and ultimately, to the exacerbation of pathology [14]. Extensive evidence links wound healing and fibrosis with Th2 differentiation, characterized by expression of cytokines IL-13 and IL-4 and protection against helminth [11–13,16].

The Th1 lineage-specific T-box transcription factor T-bet has been shown to directly repress Th17 differentiation by preventing RUNX1-mediated activation of the lineage-specific transcription factor RORγt [17]. IL-17 and IFN-γ are derived from distinct CD4^+^ T cells, and the production of each cytokine is suppressed by the other [18]. Using an *in vitro* system, Stadecker et al. have demonstrated that egg-induced Th17 cell development is driven primarily by IL-23 and IL-1β [19]. Two groups have shown that IL-17-deficient mice develop reduced liver injury compared to wild-type mice [20,21]. Previous work has suggested that ICOS is essential for Th17 cell development [18]. Spleen cells isolated from ICOS^−/−^ mice produce significantly less IL-17 than those from normal animals [22]. In contrast, ICOS stimulation of naïve, splenic T cells from normal mice increases IL-17 production [22,23]. CBA mice, a naturally high pathology strain, also displayed elevated IL-17 levels comparable to those seen in SEA/CFA-immunized BL/6 mice, and their lesion were similarly reduced by *in vivo* treatment with anti-IL-17 [24]. Taken together, these findings suggest that Th17 responses are essential for the establishment of schistosome egg-induced immunopathology and that ICOS plays an important role in the regulation of the Th17 response. However, neither the significance nor the immunopathogenesis of this pathway have been elucidated in schistosomiasis fibrosis. Here, usingthe ICOSL KO mice as a model of schistosomiasis,westudied the role of the ICOSL/ICOS interaction in the mediation of the Th17 response in host granulomatous inflammation, particularly in liver fibrosis during *S. japonicum* infection. It might reveal new therapeutic targets that interfere with Th17 cell migration or differentiation in granulomas and the subsequent fibrosis following infection with *S. japonicum*.

## Materials and Methods

### Ethics statement

Animal experiments were performed in strict accordance with the Regulations for the Administration of Affairs Concerning Experimental Animals (1988.11.1), and all efforts were made to minimize suffering. All animal procedures were approved by the Institutional Animal Care and Use Committee (IACUC) of SoochowUniversity for the use of laboratory animals (Permit Number: 2007–13).

### Mice, parasites, and infection

Female C57BL/6 WT mice (6–8 weeks old) were purchased from the Center of Comparative Medicine of Yangzhou University (Yangzhou, China). Female ICOSL KO C57BL/6 mice were purchased from Jackson Laboratory (Bar Harbor, Maine, USA). All mice were raised under specific pathogen-free conditions at the laboratory animal research facility of Soochow University (Suzhou, China). Snails (*Oncomelaniahupensis*) harboring *S. japonicum* cercariae (Chinese mainland strain) were purchased from Jiangsu Institute for Schistosomiasis Control (Wuxi, China).

For the kinetic analysis of T cell populations, cytokines and serumhyaluronic acid (HA)and hydroxyproline (HYP) titers, each mouse was infected with 14(±1) cercariae of *S. japonicum* through the abdominal skin. At 4, 7, 12, 16, and 20 weeks post-infection, five mice were randomly chosen from the infected and control groups and sacrificed for further studies.

### Splenocyte culture and cytokine detection

Single-cell splenocyte suspensions were prepared by mincing the spleens in PBS (Sigma Corporation, St. Louis, USA) containing 1% FBS (Gibco, Grand Island, NY). Red blood cells were lysed using ACK lysis buffer. Soluble egg antigens (SEA) of *S. japonicum* were purchased from Jiangsu Institute for Schistosomiasis Control (Wuxi, China). The splenocytes from *S. japonicum* infected mice were cultured in complete RPMI 1640 medium (Gibco) containing 10% FBS, 25 μg/ml of SEA, 100 U of penicillin/ml, and 0.1 mg/ml of streptomycin. Subsequently, the cells were plated in flat-bottom 96-well platesat a density of 3×10^6^ per well and cultured withcomplete media for 72 hours (h)at 37°C in 5% CO_2_ condition. Culture supernatants were collected at 4, 7, 12, 16, and 20 weeks post-infection for ELISA.

IFN-γ, IL-2, IL-12p40, IL-4, IL-17A,IL-21, TGF-β1,IL-13,IL-6 and IL-10levels in the cultured supernatant were measured by ELISA using the ELISA Ready-SET-Go kit (eBiosicence, San Diego, CA) according to the manufacturer’s protocol. The optical density (OD) of the plates was read at 450 nm using an ELISA reader (Bio-Rad mod. 550).

### Serum analysis

Serum HA and HYPtiters of C57BL/6 WT mice infected with *S. japonicum* were measured by ELISA using the ELISA Ready-SET-Go kit (eBiosicence, San Diego, CA) according to the manufacturer’s protocol. The optical density (OD) of the plates was read at 450 nm using an ELISA reader (Bio-Rad mod. 550).

### Immunohistochemistry

Livers were dissected and immediately fixed in 10% buffered formalin, and liver sections were embedded in paraffin. The immunohistochemical technique employed a two-step method (peroxidase-conjugated polymer). Endogenous peroxidases were blocked with 3% H_2_O_2_,and the non-specific binding was inhibited with 10% normal goat serum (Boster, Wuhan, China). The slides were incubated with primary antibodies diluted with PBS (1:100) at 37°C for 1 h and then at 4°Covernight. The slides were further incubated with the secondary antibodies (ChemMate Envision/HRP, rabbit/mouse detection kit, Gene-tech, Shanghai, China) at 37°C for 1 h. The reaction was then developed with DAB as the chromogen, and histological sections were counterstained with Mayer’s hematoxylin. For the negative controls, PBS was used instead of the primary antibody. In brief, five high-power fields (×400) were randomly selected and observed with a light microscope (Leica DM2500). The staining intensity and percentage of positive cells were assessed using the Leica QWin Plus software, version 3.5.1 (Leica Microsystems, Switzerland). The percentage of positively staining cells in 15 granulomas was assessed using the Leica QWin Plus software, version 3.5.1 (Leica Microsystems, Switzerland). The mean of % positively staining cells in 15 granulomas +/− SD was then calculated to assess the expression of TGF-β1(rabbitpolyclonal; 1:500; Santa Cruz,USA), IL-13(rabbitpolyclonal, 1:100, Boster, China), and the levels of the fibrosis-associated immunopathological elements MMP-9(rabbitpolyclonal, 1:150, Boster, China) and TIMP-1(rabbitpolyclonal, 1:100, Boster, China) in the liver granulomas.

### Histopathologicalstudy

Paraformaldehyde-fixed liver specimens were dehydratedin a graded alcohol series. Following xylene treatment,the specimens were embedded in paraffin blocks, cutinto 5-μm thick continuous sections, and placed on glass slides. The sections were then stained with Masson trichrome (MT) according to standardprocedures [25]. To describe and evaluate liver pathologicalchanges, a pathologist who was blinded to the researchdesign examined 10 different low-power fields of MT-stained sections (selected fields were in almostthe same location) for each mouse. In addition, the percentageof collagen calculated by a multimedia color imageanalysis system (Image-Pro Plus 6.0) was measured asa relative objective index to evaluate the degree of liver fibrosis. Each MT-stainedsection was examined at 400×magnification. Fibrotic areas were scanned and summed bythe software. The percentage of collagen was expressedas the ratio of the collagen-containing area to the wholearea [25].

Furthermore,liver samples from ICOSL knockout and C57BL/6J mice at 7, 12, 16 and 20 weeks after infection were embedded in paraffin. Liver sections were examined under a microscope to determine the pathology of egg granuloma. Individual egg granulomas were located and their maximal diameters across opposing axes (A and B) were measured under an optical microscope; the volume of granuloma was calculated based on formula V = πAB^2^/6 [26].

### Flow cytometry

Briefly, 1×10^6^ splenocytes were incubated in 100 μl of PBS. The cells were then stained with a mixture of PE- and FITC-conjugated mAbs for 30 min, washed twice, then fixed with 4% paraformaldehyde in PBS. The analysis was performed on a FACS Calibur Flow Cytometer (BD Biosciences). All procedures were performed on ice until the time of analysis. The mAbs for flow cytometry were purchased from eBioscience (San Diego, USA) and included FITC-conjugated anti-CD4, FITC-conjugated anti-CD19, PE-conjugated anti-ICOS, PE-conjugated anti-ICOSL, PE-conjugated anti-RORγtand PE-conjugated anti-IL-17R.

### Statistical analysis

All statistical analyses were performed by SPSS13.0 Data Editor (SPSS Inc., Chicago, IL, USA). The differences of the data between all the groups were compared by two-way ANOVA followed by Tukey’s multiple comparison test. Spearman’s rank correlation was used for correlation analyses. Differences with *P*<0.05 were considered statistically significant. Each individualexperiment was conducted with groups of 5 mice and repeatedat least twotimes.

## Results

### Reduced Th17 responses in ICOSL KO mice infected with *S. japonicum*

**Figure 1:**
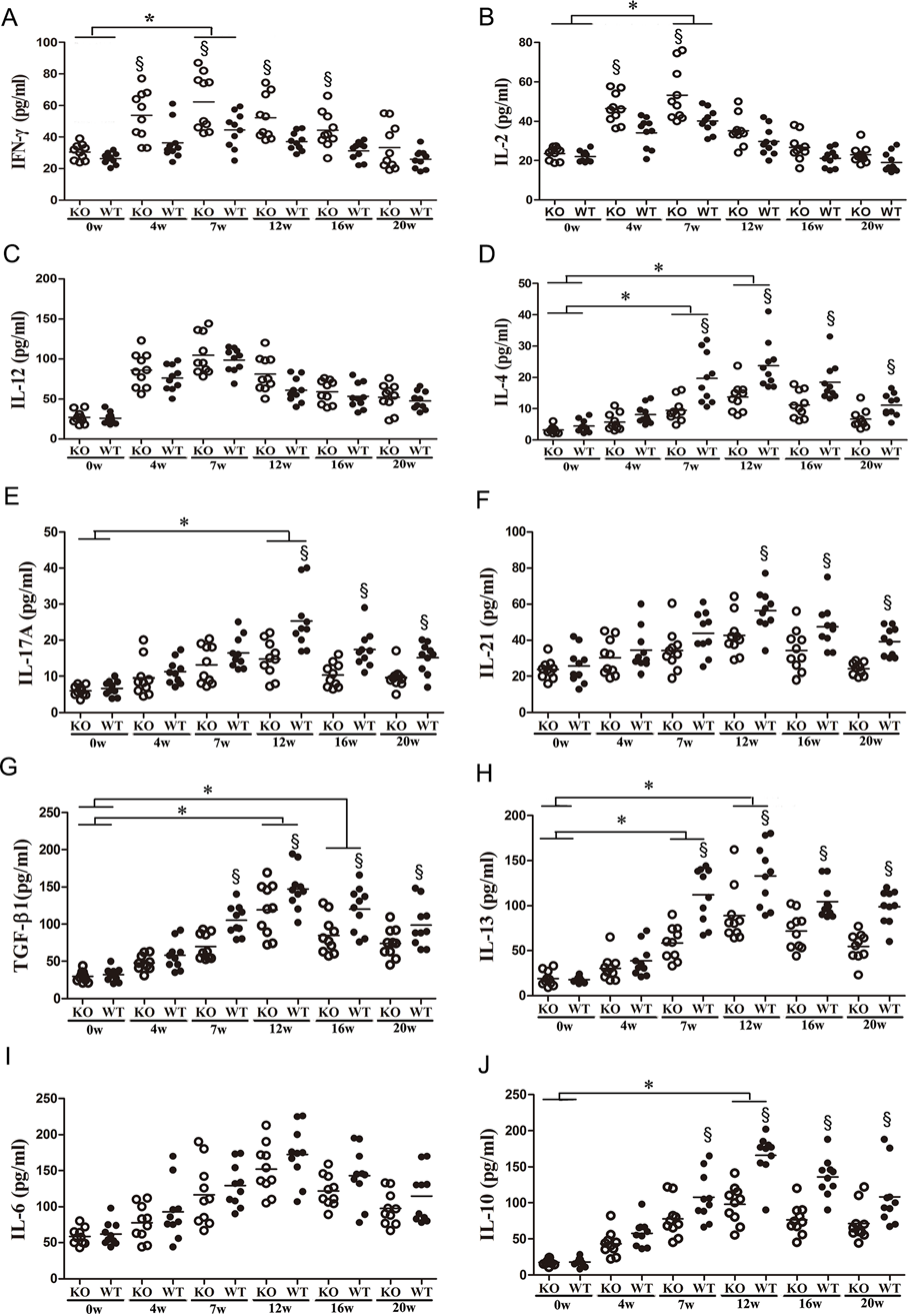
Kinetics of cytokine production by splenocytes from ICOSL KO and WT mice infected with *Schistosoma japonicum*. Female ICOSL KO and WT mice were infected with 14±1 cercariae of *S. japonicum* per mouse. A total of 30 mice(five mice per group)were randomly chosen and sacrificed at 0 (before infection), 4, 7, 12, 16, or 20 weeks post-infection. Single-cell splenocyte suspensions were prepared and then cultured in the presence of SEA. The culture supernatants were collected after 72 h of incubation for the detection of IFN-γ (A), IL-2 (B),IL-12 (C), IL-4 (D), IL-17A (E),IL-21 (F), TGF-β1 (G), IL-13 (H),IL-6 (I) and IL-10 (J) by ELISA. Open symbols represent ICOS KO mice and closed symbols represent WT mice. Data shown are pooled from two independent experiments, symbols denotes individual mice and lines denote mean values. §Significant genotype effect and * significant time effect (*P*< 0.05, Tukey HSD, ANOVA performed using combined data from two separate experiments).

To elucidate the role of ICOSL/ICOS signaling in modulating the pattern of cytokine production in the ICOSL KO and WT C57BL/6 control strains, the mice were infected with *S. japonicum* (14±1 cercariae) and euthanized at 0 (before infection), 4 (early stage), 7 (acute stage), 12(chronic stage) or 16 (advanced stage) weeks post-infection. Splenocytes were cultured in the presence of SEA for 72 h, and cytokines were measured in the culture supernatants by ELISA. The results showed that the production of IFN-γ, IL-2 and IL-12 (Th1-related cytokines) were elevated at 4 weeks post-infection and peaked at 7 weeks post-infection(Fig. 1A, 1B, 1C). When the disease progressed from the acute to the chronic phase, the expression levels of Th1-related cytokines started to decrease, while the production of IL-4/IL-6/IL-10(Th2-related cytokines) (Fig. 1D, 1I, 1J) and IL-17A/IL-21 (Th17-related cytokines) (Fig. 1E, 1F) increased. The levels of Th2- and Th17- related cytokines peaked at 12 weeks post-infection before decreasing gradually. The production of IL-17A/IL-21 by ICOSL KO mice was significantly lower than that byWT controls (Fig. 1E, 1F). In contrast, the production of IFN-γby ICOSL KO mice was significantly higher than that byWT controls (Fig. 1A).

The production of anti-inflammatory cytokine, TGF-β1, and the classic pro-fibrotic cytokine IL-13 by whole spleen cells were measured following infection. The results showed that ICOSL KO mice produced significantly lower amounts of TGF-β1 and IL-13 than those of the WT controls (Fig. 1G, 1H).

Then, we determined the HA and HYP titers in C57BL/6 mice following *S. japonicum* infection and analyzed the correlation between the HA/HYPtiters and the IL-4/IL-13/TGF-β1/IL-10/IL-17A levels (S1 Fig.). The IL-4/IL-13/TGF-β1 levels were positively correlated with the HA/HYP titers in C57BL/6 mice following *S. japonicum* infection (*P*<0.0001) (S2 Fig.). Importantly, the IL-17A levels were also positively correlated with the HA/HYP titers in C57BL/6 mice following *S. japonicum* infection (*P*<0.01 or *P*<0.05) (S2 Fig.). The data suggest that ICOSL/ICOS interactions play a key role in establishing the pattern of the cytokine response to *S. japonicum* infection.

### Down-regulation of ICOS and RORγt expression by ICOSL-KO mice andthe positive correlation between RORγtand HA/HYP titers in mice infected with *S. japonicum*

**Figure 2:**
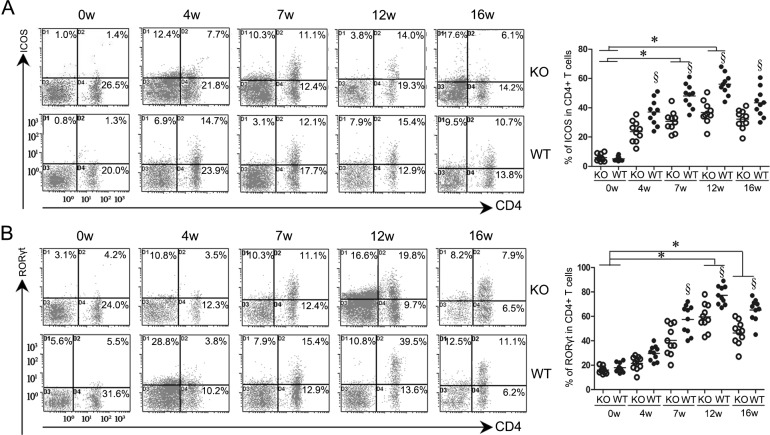
Lower expression of ICOS and RORγt on the surface of CD4^+^ T cells in ICOSL KO mice infected with *Schistosoma japonicum*. The mice were infected with 14±1 cercariae of *S. japonicum*. A total of 25 mice (five mice per group)were randomly chosen and sacrificed at 0 (before infection), 4, 7, 12, or 16weeks post-infection. The spleen cells were collected and stained for CD4 and ICOS(A) and RORγt(B) as described in the Materials and Methods. All density plot charts were based on 10^4^ cells, satisfying a gate set on the forward versus side light scatter that defined spleen lymphocytes. The percentages of double-positive cells are indicated in the upper right of each chart. Open symbols represent ICOS KO mice and closed symbols represent WT mice. Data shown are pooled from two independent experiments, symbols denotes individual mice and lines denote mean values. §Significant genotype effect and * significant time effect (*P*< 0.05, Tukey HSD, ANOVA performed using combined data from two separate experiments).

**Figure 3:**
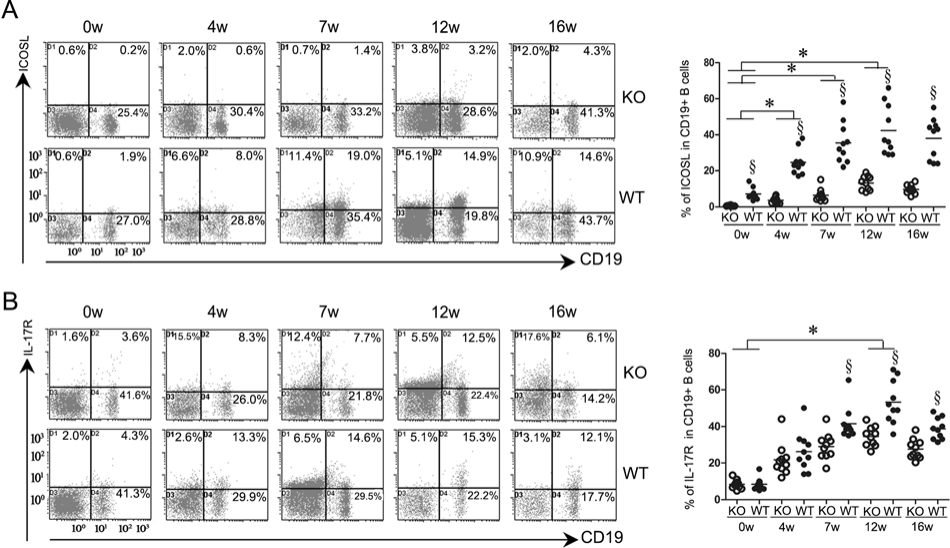
ICOSL KO mice infected with *Schistosoma japonicum* produced lower levels of ICOSL and IL-17R on CD19^+^ B cells than those of infected WT mice. CD19^+^B cells isolated from the spleens were stained for CD19 and were then stained for ICOSL (A) and IL-17R(B) as described in the Materials and Methods. All density plot charts were based on 10^4^ cells, satisfying a gate set on the forward versus side light scatter that defined spleen lymphocytes. Open symbols represent ICOS KO mice and closed symbols represent WT mice. Data shown are pooled from two independent experiments, symbols denotes individual mice and lines denote mean values. §Significant genotype effect and * significant time effect (*P*< 0.05, Tukey HSD, ANOVA performed using combined data from two separate experiments).

The expression levels of ICOS and ICOSL in splenocytes of the WT mice were elevated at 4 weeks post-infection and peaked at 12 weeks before decreasing gradually (Figs. 2A, 3A). Relatively small changes in ICOS expression were observed in CD4^+^ T cells of ICOSL KO mice infected with *S. japonicum*. In contrast, the WT mice showed a comparatively considerable increase in ICOS expression after infection. The data also showed that the expression of ICOS was significantly down-regulated in ICOSL KO mice compared to WT controls at 4, 7, 12, and 16 weeks post-infection (Fig. 2A). As expected, the expression of ICOSL was extremely low in ICOSL KO mice at all time points after *S. japonicum* infection (*P*<0.05) (Fig. 3A). ICOSL KO mice also exhibited decreased levels of RORγtand IL-17Rafter 4 weeks post-infection (Figs. 2B and 3B).

We then analyzed the correlation between the RORγt levels and the HA/HYP titers. The RORγt levels were positively correlated with the HA and HYP titers in C57BL/6 mice following *S. japonicum* infection (*P*<0.0001) (S3 Fig.).

These data suggest thatICOSL/ICOS might be required for expanding IL-17-producing cells in mice following *S. japonicum* infection and Th17 responses are involved in the pathogenesis of fibrosis.

### Reduced progression of fibrogenesis in ICOSL KO mice

**Figure 4:**
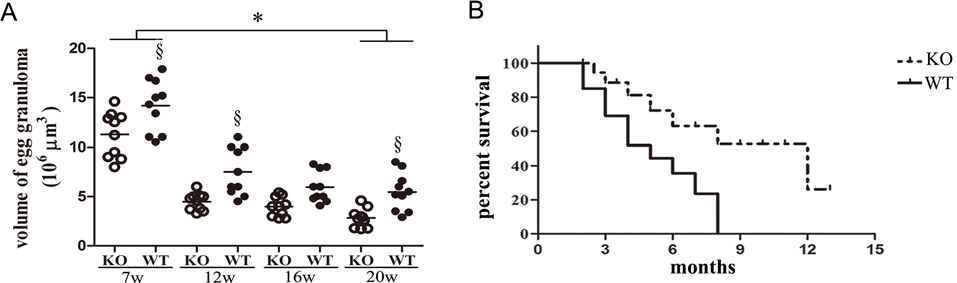
Reduced granulomatous formation and improved survival in the ICOSLKO mice. (A). Liver samples from ICOSL knockout and C57BL/6J mice 7, 12, 16 and 20 weeks post-infection with *Schistosoma japonicum* were embedded in paraffin. Liver sections were examined under a microscope to determine the pathology of egg granuloma; individual egg granulomas were located and their maximal diameters across opposing axes (A and B) were measured under an optical microscope; the volume of granuloma was calculated based on formula V = πAB^2^/6 [26]. Open symbols represent ICOS KO mice and closed symbols represent WT mice. There were 5 mice at each time point and 10granulomas in each mouse were measured at each time point. Symbols denotes the mean of 10granulomas in each mouse and lines denote mean values. §Significant genotype effect and * significant time effect (*P*< 0.05, Tukey HSD, ANOVA performed using combined data from two separate experiments). (B). Kaplan-Meier survival analysis of mice inoculated with *S. japonicum*(ICOSL KO vs WT, n = 20, Log rank test *P* = 0.017). The survival analysis data are representative of three independent experiments with similar results.

Histological examination of granulomas in mice infected with *S. japonicum* showed that granulomas were found in mouse livers 7 weeks post-infection (Fig. 4A). The volume of the granuloma was the largest at that time point (Fig. 4A). Twelve weeks after the infection, the formation of granuloma began to reduce (Fig. 4A). Comparing the volume of granulomas in the ICOSL KO and wild type mice at 7, 12, 16 and 20 weeks after the infection showed that the size of granulomas were significantly smaller in the ICOSL KO mice than those of the WT mice(*P*<0.05) (Fig. 4A). Furthermore, ICOSL KO mice showed improved survival compared to WT mice *(P* = 0.017) (Fig. 4B).

**Figure 5:**
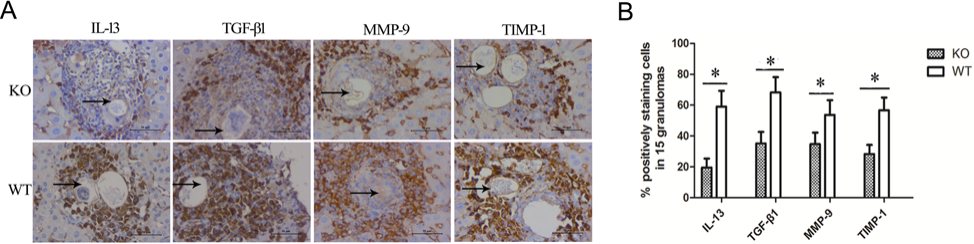
Expression of fibrosis-related factors in ICOSL KO and WT mice. (A). The areas of egg granulomas or fibrosis were chosen, and a two-step immunohistochemical method (peroxidase-conjugated polymer) was used on paraffin section of liver as described in the Materials and Methods. The dark brown area represents positive particles and arrow shows eggs of *S. japonicum* (original magnification×400). (B). The percentage of positive staining cells in 15 granulomas was calculated to assess the expression of IL-13, TGF-β1, MMP-9 and TIMP-1 on inflammatory cells of the granulomas in ICOSL KO mice at 12 weeks (IL-13 and TGF-β1) or 16 weeks (MMP-9 and TIMP-1) post-infection. Each bar represents mean of % positively staining cells in 15 granulomas +/− SD of 5 mice per group from two independent experiments.*P<0.05 between ICOSL KO and WT mice groups, Student’s t-test.

Along with more extensive size of granulomas in the liver, the expression of TGF-β1 and IL-13 was significantly increased in murine schistosomiasis(Fig. 5B). Many cells were stained dark yellow in the slides of liver tissue, mainly around granuloma inflammatory cellsand the portal area cells around areas of fibrosis (Fig. 5A). Liver cells around granulomas also showed strong expression of TGF-β1 and IL-13. The expression levels of TGF-β1 and IL-13 in *S. japonicum* infected ICOSL KO mice were lower than those in *S. japonicum* infected WT mice at 12 weeks post-infection (*P*<0.05) (Fig. 5B). These observations were consistent with analysis of cytokines in the supernatants of SEA-stimulated splenocytes (Fig. 1G, 1H). In addition, we observed lower levels of the fibrosis-associated immunopathologic elements MMP-9 and TIMP-1 in the ICOSL KO mice compared to the controls at the advanced stage of infection (16 weeks) (Fig. 5B).

**Figure 6:**
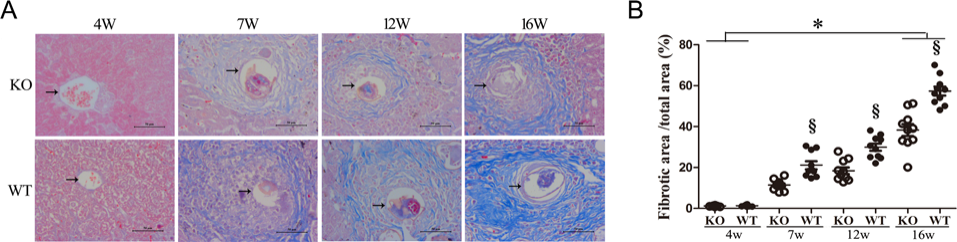
Reduced liver fibrogenesis of schistosomiasis in ICOSL KO mice. The degree of hepatic fibrosis was accessed by staining collagen using Masson Trichrome method. Each mouse was sacrificed at 4, 7, 12 and 16 weeks post-infection and liver tissue was examined for collagen expression. (A). Liver fibrosis as determined by Masson staining (original magnification×400). The blue area represents fibrillar collagen and arrow shows eggs of *S. japonicum*. (B). The fibrosis area of the section was quantified using image-Pro Plus 6.0 software, the ratio of collagen area and total area (%) was counted and compared between ICOSL KO and WT mice. Data shown are pooled from two independent experiments. Symbols denotes the mean of ten different field of individual liver sample and lines denote mean values. Open symbols represent ICOS KO mice and closed symbols represent WT mice. §Significant genotype effect and * significant time effect (*P*< 0.05, Tukey HSD, ANOVA performed using combined data from two separate experiments).

Masson trichrome stained sections of liver showed typical pathological characteristics of liver of schistosomiasis with remarkable acute granuloma formation and subsequent liver fibrosis from week 7 through week 16 (Fig. 6A). Moreover, analysis of Mason trichrome staining showed that the ICOSL KO group developed mild hepatic fibrosis and reduced collagen production compared to WT mice (Fig. 6B).

These data indicate that blockade of ICOSL/ICOS interaction might decrease schistosomiasis-induced immunopathology and fibrogenesis by suppressing Th17 and Th2 generation.

## Discussion

Schistosomiasis is a parasitic disease that has a devastating impact on both humans and animals [9]. Both humoral immunity and cellular immunity are involved in the formation and development of hepatic egg granuloma [9,14]. The full activation and differentiation of T cells into Th1, Th2 or Th17 cells requires co-stimulatory molecules and cytokines [1–4]. ICOS has also been implicated in chronic inflammation and is critical for Th17 cell development [21]. By use of anti-ICOS mAbs, the blockade of ICOS during the effector phase of EAE has been shown to abrogate disease, whereas blockade during priming does not; this suggests an important role for ICOS in Th17 effector responses [27]. The ICOSL/ICOS pathway plays different roles in different models of autoimmune and infectious disease [28–31]. ICOS knockout miceareincapable of controlling viral or worm infections owing toimpaired Th1 and Th2 responses, respectively [28]. Administration of blocking anti-ICOSL mAbs results in a striking reduction in the development of experimental rheumatoid arthritis and lupus nephritis, which correlates with reduced T follicular helper (Tfh) differentiation and germinal center formation [29].

Although several studies have focused on the contribution of Th2 cells to chronic inflammatory disease and fibrosis, which is characterized by the cytokines IL-4 and IL-13 [11,12,16,32], CD4^+^ IL-17-secreting T cells have been shown to contribute to pathology in some models of liver fibrosis. In patients with idiopathic pulmonary fibrosis (IPF), IL-17A cooperates with and cross-regulates IL-1β, and the interaction of these cytokines contributes critically to neutrophil recruitment and lung fibrosis and possibly to liver fibrosis [33–35]. IL-17A is also elevated in IPF patients [35].

The analysis of cytokines in the supernatants of SEA-stimulated splenocytes (Fig. 1) showed that ICOSL KO mice produced higher levels of IFN-γ but lower levels of IL-17A than WT mice. Usingthe IL-17^−/−^ and IFN-γ^−/−^ mouse model, Rutitzky et al. [14] demonstrated that severe immunopathology in murine schistosomiasis is primarily due to IL-17 and is regulated by IFN-γ. IL-17^−/−^ mice demonstrated significantly reduced immunopathology, despite increased levels of IFN-γ, while IFN-γ^−/−^ mice displayed markedly elevated immunopathology correlating with increased levels of IL-17A [14]. As shown in Fig. 1F, ICOSL KO mice displayed reduced levels of IL-21 compared to WT mice, particularly at 7 weeks post-infection. As an autocrine regulator of Th17 cell development, IL-21 plays a key role in inducing the differentiation of Th17 cells and suppressing the Th1 response [36,37]. Also, it is well known that the secretion of IL-21 is restricted mainly to Tfh cells and Th17 cells, and IL-21 is associated with Tfh cells as well as IL-17 responses [38]. Therefore, eliminating ICOSL has shown defective ICOSL/ICOS interactions in the ICOSL KO mice infected with *S. japonicum*, leading to reduced levels of IL-21, which may have decreased the Tfh and B cell responses, and thus provide one explanation for the down-regulation of the Th17 response associated with IL-21 and ICOSL/ICOS interactions. In addition, the ICOSL KO mice had elevated IL-2 levels (Fig. 1B), and this cytokine has been shown to elevate Th1 responses and suppress Th17 responses [36]. The results (Fig. 1G, 1H) also showed significantly reduced levels of TGF-β1 and IL-13 in the supernatant of SEA-stimulated splenocytes at both the acute and advanced stages. Researchers have demonstrated that TGF-β1 plays a predominant role in the differentiation of Th17 cells and limits Th1 and Th2 responses [15,36–37,39–43]. Our data further indicated that blocking ICOSL/ICOS signaling might result in defective differentiation and expansion of Th17 cells following *S. japonicum* infection through the down-regulation of TGF-β1.

The observation that ICOS was down-regulated in CD4^+^ T cells of ICOSL KO mice indicates a critical role for ICOSL/ICOS signaling in the expression of ICOS following *S. japonicum* infection.

As shown in Fig. 2B, ICOSL KO mice exhibited significantly lower levels of RORγt at the chronic stages compared with WT controls. RORγt is considered to be dispensable in the development of Th17 cells but is required for the full differentiation of naïve CD4^+^ T cells into Th17 cells [37,39–41]. These results indicated that ICOSL/ICOS interactions may promote the differentiation and expansion of Th17 cells following infection with *S. japonicum*.

Meanwhile, the percentages of RORγt^+^ cells in CD4^+^ T cells were positively correlated with the HA and HYP titers in mice infected with *S. japonicum*(S3 Fig.). Serum HA and HYPare good markers for the initial phase of hepatic fibrosis and it was able to assess severity of liver disease in schistosomiasis [6,44,45].

To further understand how the ICOSL/ICOS signal contributes to the development of severe hepatic fibrogenesis after *Schistosoma* infection, paraffin sections of liver from infected mice were evaluated by immunohistochemistry. Consistent with results of the cytokine analysis (Fig. 1), IL-13 and TGF-β1 werealso shown to be markedly reduced in ICOSL KO mice compared to the WT controls at 12 weeks post-infection (P<0.001) (Fig. 5). IL-13 is a potent inducer of MMP-9 and TGF-β1 [46,47]. TGF-β1, a known mediator of fibrosis, is the most widely studied cytokine in the context of fibrogenesis [46]. It has been suggested that IL-13 mediates its effects by regulating the production and activation of TGF-β1 [47]. The data presented hereinindicate that the neutralization of ICOSL affords protection against the development of severe hepatic granulomatous inflammation, particularly fibrosis induced by the immune response to egg Ags. The fibrotic tissue of ICOSL KO mice also contained lower levels of MMP-9 and TIMP-1 than those of WT mice (Fig. 5). Furthermore, the ICOSL KO mice exhibited lower fibrillar collagen expression around *S. japonicum* eggs (Fig. 6) and improved survival ofmice infectedwith *S. japonicum* than that of WT mice (Fig. 4B). These results provide further evidence of a down-regulated granulomatous immune response/fibrosis synthesis in ICOSL KO mice, due in part to a defect in the expansion of the differentiated Th17 cell population, but also IL-13, TGF-β1 and IL-4.

Studies with other systems have shown that ICOS expression persists at high levels on Th2 cells[48]. Furthermore, disruption of ICOSL/ICOS pathway has been found to be beneficial when treating diseases dominated by Th2-type cytokine production,such as asthma [49,50]. All these studies are in line with ours; however, ICOS deficiency or blocking ICOS has been reported to no change or increase in the size of egg induced granulomas during *S. mansoni* infection[51,52]. The results of the current study contradict these studies, which may be due to the different *Schistosoma* species or different mice strains used. It appears that the ultimate effects of the ICOSL/ICOS pathway is dependent on the particular model of study.

All of these results suggest that IL-17-producing cells could contribute to the hepatic granulomatous inflammation and subsequent fibrosis in addition to the Th1, Th2 and Th17 associated cytokines. Also, a clearly positive correlation between the presence of IL-17-producing cells and ICOS expression in ICOSL KO mice was observed, which suggested that Th17 cells were involved in the pathological tissue remodeling in liver fibrosis induced by schistosomiasis. The well-understood Th2 response in previous studies [53–55] in combination with our present studies of correlation betweenTh2/Th17 and fibrosis (S1, S2, S3 Figs.), indicated that both Th2 and Th17 responses could play animportant role in pathology/mortality including the fibrosis in schistosomiasis.

In summary, our findings indicated that Th17 responses is one of the promotor driving fibrosis in schistosomiasis in addition to Th2 responses, and emphasizedthat ICOSL/ICOS signaling mediates the IL-17-producing CD4^+^ T cell response which could contribute to severe hepatic granulomatous inflammation and subsequent fibrosis via Th17. This study further clarifies the immune regulatory mechanism of fibrosis and sheds light on the understanding of the immunopathogenesis of *Schistosoma* induced fibrosis.

## Supporting Information

S1 Fig The analysis of correlation betweenTh2/Th17 responses and fibrosis. The linear relationship of IL-4 (A: R = 0.5858, ***P = 0.0007), IL-13 (C:R = 0.7432, ***P<0.0001), TGF-β1 (E:R = 0.6257, ***P = 0.0002), IL-10 (G:R = 0.3548, *P* = 0.0543), IL-17A (I:R = 0.4998, **P = 0.0049) and HA. The linear relationship of IL-4 (B:R = 0.7296, ***P<0.0001), IL-13 (D:R = 0.7732, ***P<0.0001), TGF-β1 (F:R = 0.7462, ***P<0.0001), IL-10 (H: R = 0.3171, *P* = 0.0877), IL-17A (J:R = 0.4241, *P = 0.0195) and HYP. The results are representative of three independent experiments with similar results, which arefrom fiveindependent mice in each group at 6 time points (0, 4, 7, 12, 16, 20 weeks).
(TIF)

S2 Fig The comparison of Spearman r from the analysis of correlation. (A). The Spearman r from the analysis of correlation between IL-4/IL-13/TGF-β1/IL-10/IL-17A and HA. (B). The Spearman r from the analysis of correlation between IL-4/IL-13/TGF-β1/IL-10/IL-17A and HYP. *P<0.05, **P<0.01, and ***P<0.001 between cytokine and HA/HYP, Spearman’s rank.
(TIF)

S3 Fig The analysis of correlation between the RORγt levels and fibrosis. (A). The linear relationship of RORγt^+^ cells in CD4^+^ T cellsand HA (r = 0.8653, ***P<0.0001). (B). The linear relationship of RORγt^+^ cells in CD4^+^ T cellsand HYP (r = 0.7292, ***P<0.0001). The results are representative of three independent experiments with similar results, which are from five independent mice in each group at 5 time points (0, 4, 7, 12, 16 weeks).
(TIF)

## References

[1] Sharpe AH (2009) Mechanisms of costimulation.Immunol Rev 229: 5–11. 10.1111/j.1600-065X.2009.00784.x 19426211 PMC2928676

[2] Hutloff A, Dittrich AM, Beier KC, Eljaschewitsch B, Kraft R, et al.(1999) ICOS is an inducible T-cell costimulator structurally and functionally related to CD28. Nature 397: 263–266. 10.1038/16717 9930702

[3] Simpson TR, Quezada SA, Allison JP (2010) Regulation of CD4 T cell activation and effector function by inducible costimulator (ICOS).Curr Opin Immunol 22:326–332. 10.1016/j.coi.2010.01.001 20116985

[4] Dong C, Juedes AE, Temann UA, Shresta S, Allison JP, et al. (2001) ICOS co-stimulatory receptor is essential for T-cell activation and function. Nature 409: 97–101. 10.1038/35051100 11343121

[5] Mak TW, Shahinian A, Yoshinaga SK, Wakeham A, Boucher LM, et al. (2003) Costimulation through the inducible costimulator ligand is essential for both T helper and B cell functions in T cell-dependent B cell responses. Nat Immunol 4: 765–772. 10.1038/ni947 12833154

[6] Popper H, Kent G (1975) Fibrosis in chronic liver disease. Clin Gastroenterol 4: 315–332. 1092488

[7] Kaviratne M, Hesse M, Leusink M, Cheever AW, Davies SJ, et al. (2004) IL-13 activates a mechanism of tissue fibrosis that is completely TGF-beta independent. J Immunol 173:4020–4029. 15356151 10.4049/jimmunol.173.6.4020

[8] Albanis E, Safadi R, Friedman SL (2003) Treatment of hepatic fibrosis: almost there. Curr Gastroenterol Rep 5: 48–56. 10.1007/s11894-003-0009-7 12530948

[9] Pearce EJ, MacDonald AS (2002) The immunobiology of schistosomiasis. Nat RevImmunol 2: 499–511. 10.1038/nri843 12094224

[10] Gause WC, Urban JF, Stadecker MJ (2003) The immune response to parasitic helminths: insights from murine models. Trends Immunol 24: 269–277. 10.1016/S1471-4906(03)00101-7 12738422

[11] Henderson NC, Iredale JP (2007) Liver fibrosis: cellular mechanisms of progression and resolution. Clin Sci (Lond) 112: 265–280. 10.1042/CS20060242 17261089

[12] Holt AP, Salmon M, Buckley CD, Adams DH (2008) Immune interactions in hepatic fibrosis. Clin Liver Dis 12: 861–882. 10.1016/j.cld.2008.07.002 18984471 PMC2605646

[13] Wynn TA (2008) Cellular and molecular mechanisms of fibrosis. J Pathol 214: 199–210. 10.1002/path.2277 18161745 PMC2693329

[14] Rutitzky LI, Stadecker MJ (2011)Exacerbated egg-induced immunopathology in murine Schistosoma mansoni infection is primarily mediatedby IL-17 and restrained by IFN-γ.Eur J Immunol 41: 2677–2687. 10.1002/eji.201041327 21660933 PMC3679923

[15] McGeachy MJ, Bak-Jensen KS, Chen Y, Tato CM, Blumenschein W, et al.(2007) TGF-beta and IL-6 drive the production of IL-17 and IL-10 by T cells and restrain T(H)-17 cell-mediated pathology. Nat Immunol 8: 1390–1397. 10.1038/ni1539 17994024

[16] Sandler NG, Mentink-Kane MM, Cheever AW, Wynn TA (2003) Global gene expression profiles during acute pathogen-induced pulmonary inflammation reveal divergent roles for Th1 and Th2 responses in tissue repair. J Immunol 171: 3655–3667. 14500663 10.4049/jimmunol.171.7.3655

[17] Lazarevic V, Chen X, Shim JH, Hwang ES, Jang E, et al. (2011) T-bet represses T(H)17 differentiation by preventing Runx1-mediated activation of the gene encoding RORγt. Nat Immunol 12: 96–104. 10.1038/ni.1969 21151104 PMC3077962

[18] Park H, Li Z, Yang XO, Chang SH, Nurieva R, et al. (2005) A distinct lineage of CD4 T cells regulates tissue inflammation by producing interleukin 17. Nat Immunol 6: 1133–1141. 10.1038/ni1261 16200068 PMC1618871

[19] Shainheit MG, Smith PM, Bazzone LE, Wang AC, Rutitzky LI, et al. (2008) Dendritic cell IL-23 and IL-1 production in response to schistosome eggs induces Th17 cells in a mouse strain prone to severe immunopathology. J Immunol 181: 8559–8567. 19050275 10.4049/jimmunol.181.12.8559 PMC2663362

[20] Nagata T, McKinley L, Peschon JJ, Alcorn JF, Aujla SJ, et al. (2008) Requirement of IL-17RA in Con A induced hepatitis and negative regulation of IL-17 production in mouse T cells. J Immunol 181: 7473–7479. 19017936 10.4049/jimmunol.181.11.7473

[21] Lafdil F, Wang H, Park O, Zhang W, Moritoki Y, et al. (2009) Myeloid STAT3 inhibits T Cell-mediated hepatitis by regulating T helper 1 cytokine and interleukin-17 production. Gastroenterology 137: 2125–2135. 10.1053/j.gastro.2009.08.004 19686746 PMC2789897

[22] Nurieva RI, Treuting P, Duong J, Flavell RA, Dong C (2003) Inducible costimulator is essential for collagen-induced arthritis. J Clin Invest 111: 701–706. 10.1172/JCI17321 12618524 PMC151904

[23] Nurieva RI (2005) Regulation of immune and autoimmune responses by ICOS-B7h interaction. Clin Immunol 115: 19–25. 10.1016/j.clim.2005.02.010 15870016

[24] Rutitzky LI, Lopes da Rosa JR, Stadecker MJ (2005) Severe CD4 T cell-mediated immunopathology in murine schistosomiasis is dependent on IL-12p40 and correlates with high levels ofIL-17. J Immunol 175: 3920–3926. 16148138 10.4049/jimmunol.175.6.3920

[25] Chen BL, Zhang GY, Yuan WJ, Wang SP, Shen YM, et al. (2011) Osteopontin expression is associated with hepatopathologic changes in Schistosoma japonicum infected mice. World J Gastroenterol 17:5075–5082. 10.3748/wjg.v17.i46.5075 22171141 PMC3235590

[26] He Y, Shi F, Clarde WR, HSü SY, HSü HF (1994) Schistosoma japonicum: size of egg granulomas in vaccinated and non-vaccinated hosts as observed in bovines. Chin J Parasitol Parastic Dis 12: 196–199. 7867155

[27] Rottman JB, Smith T, Tonra JR, Ganley K, Bloom T, et al. (2001) The costimulatory molecule ICOS plays an important role in the immunopathogenesis of EAE.Nat Immunol 2: 605–611. 10.1038/89750 11429544

[28] Kopf M, Coyle AJ, Schmitz N, Barner M, Oxenius A, et al. (2000) Inducible costimulatorprotein (ICOS) controls T helper cell subset polarization after virus and parasite infection. J Exp Med 192: 53–61. 10.1084/jem.192.1.53 10880526 PMC1887704

[29] Hu YL, Metz DP, Chung J, Siu G, Zhang M (2009) B7RP-1 blockade ameliorates autoimmunity through regulation of follicular helper T cells. J Immunol 182: 1421–1428. 19155489 10.4049/jimmunol.182.3.1421

[30] Hawiger D, Tran E, Du W, Booth CJ, Wen L, et al. (2008) ICOS mediates the development of insulin-dependent diabetes mellitus in nonobese diabetic mice. J Immunol 180: 3140–3147. 18292537 10.4049/jimmunol.180.5.3140

[31] Clay BS, Shilling RA, Bandukwala HS, Moore TV, Cannon JL, et al. (2009) Inducible costimulator expression regulates the magnitude of Th2-mediated airway inflammation by regulating the number of Th2 cells. PLoS ONE 4: e7525 10.1371/journal.pone.0007525 19888475 PMC2768787

[32] Seki E, De Minicis S, Gwak GY, Kluwe J, Inokuchi S, et al. (2009) CCR1 and CCR5 promote hepatic fibrosis in mice. J Clin Invest 119: 1858–1870. 19603542 10.1172/JCI37444 PMC2701864

[33] Gasse P, Mary C, Guenon I, Noulin N, Charron S, et al. (2007) IL-1R1/MyD88 signaling and the inflammasome are essential in pulmonary inflammation and fibrosis in mice. J Clin Invest 117: 3786–3799. 10.1172/JCI32285 17992263 PMC2066195

[34] Seki E, De Minicis S, Osterreicher CH, Kluwe J, Osawa Y, et al. (2007) TLR4 enhances TGF-beta signaling and hepatic fibrosis. Nat Med 13: 1324–1332. 10.1038/nm1663 17952090

[35] Wilson MS, Madala SK, Ramalingam TR, Gochuico BR, Rosas IO, et al. (2010) Bleomycin and IL-1beta-mediated pulmonary fibrosis is IL-17A dependent. J Exp Med 207: 535–552. 10.1084/jem.20092121 20176803 PMC2839145

[36] Harrington LE, Hatton RD, Mangan PR, Turner H, Murphy TL, et al.(2005) Interleukin 17-producing CD4^+^ effector T cells develop via a lineage distinct from the T helper type 1 and 2 lineages. Nat Immunol 6: 1123–1132. 10.1038/ni1254 16200070

[37] Bi Y, Liu G, Yang R (2007) Th17 cell induction and immune regulatory effects. J Cell Physiol 211: 273–278. 17311299 10.1002/jcp.20973

[38] Pallikkuth S, Pahwa S (2013) Interleukin-21 and T follicular helper cells in HIV infection: research focus and future perspectives.Immunol Res 57:279–291. 10.1007/s12026-013-8457-0 24242760 PMC6510583

[39] Harrington LE, Mangan PR, Weaver CT (2006) Expanding the effector CD4 T-cell repertoire: the Th17 lineage. Curr Opin Immunol 18: 349–356. 10.1016/j.coi.2006.03.017 16616472

[40] Dong C (2008) Th17 cells in development: an updated view of their molecular identity and genetic programming. Nat Rev Immunol 8: 337–348. 10.1038/nri2295 18408735

[41] McGeachy MJ, Cua DJ (2008) Th17 cell differentiation: the long and winding road. Immunity 28: 445–453. 10.1016/j.immuni.2008.03.001 18400187

[42] Park IK, Shultz LD, Letterio JJ, Gorham JD (2005) TGF-β1 inhibits T-bet induction by IFN-γ in murine CD4+ T cells through the protein tyrosine phosphatase Src homology region 2 domain-containing phosphatase-1. J Immunol 175: 5666–5674. 16237056 10.4049/jimmunol.175.9.5666

[43] Matsuzaki G, Umemura M (2007) Interleukin-17 as an effector molecule of innate and adaptive immunity against infections. Microbiol Immunol 51: 1139–1147. 10.1128/IAI.01951-14 18094532

[44] Köpke-Aguiar LA, Martins JR, Passerotti CC, Toledo CF, Nader HB, et al. (2002) Serum hyaluronic acid as a comprehensive marker to assess severity of liver disease in schistosomiasis. Acta Trop 84:117–126. 10.1016/S0001-706X(02)00136-5 12429428

[45] El-Lakkany NM, Hammam OA, El-Maadawy WH, Badawy AA, Ain-Shoka AA, et al. (2012) Anti-inflammatory/anti-fibrotic effects of the hepatoprotectivesilymarin and the schistosomicide praziquantel against Schistosoma mansoni-induced liver fibrosis. Parasit Vectors 5: 9 10.1186/1756-3305-5-9 22236605 PMC3398291

[46] Lee CG, Homer RJ, Zhu Z, Lanone S, Wang X, et al. (2001) Interleukin-13 induces tissue fibrosis by selectively stimulating and activating transforming growth factor beta(1). J Exp Med 194: 809–822. 10.1084/jem.194.6.809 11560996 PMC2195954

[47] Zhu Z, Homer RJ, Wang Z, Chen Q, Geba GP, et al. (1999) Pulmonary expression of interleukin-13 causes inflammation, mucus hypersecretion, subepithelial fibrosis, physiologic abnormalities, and eotaxin production. J Clin Invest 103: 779–788. 10.1172/JCI5909 10079098 PMC408149

[48] McAdam AJ, Chang TT, Lumelsky AE, Greenfield EA, Boussiotis VA, et al. (2000) Mouse inducible costimulatory molecule (ICOS) expression is enhanced by CD28 costimulation and regulates differentiation of CD4+ T cells. J Immunol 165:5035–5040. 11046032 10.4049/jimmunol.165.9.5035

[49] Gonzalo JA, Tian J, Delaney T, Corcoran J, Rottman JB, et al. (2001) ICOS is critical for T helper cell-mediated lung mucosal inflammatory responses. Nat Immunol 2:597–604. 10.1038/89739 11429543

[50] Tesciuba AG, Subudhi S, Rother RP, Faas SJ, Frantz AM, et al. (2001) Inducible costimulator regulates Th2-mediated inflammation, but not Th2 differentiation, in a model of allergic airway disease. J Immunol 167:1996–2003. 11489981 10.4049/jimmunol.167.4.1996

[51] Rutitzky LI, Ozkaynak E, Rottman JB, Stadecker MJ (2003) Disruption of the ICOS-B7RP-1 costimulatory pathway leads to enhanced hepatic immunopathology and increased gamma interferon production by CD4 T cells in murine schistosomiasis. Infect Immun 71: 4040–4044. 10.1128/IAI.71.7.4040-4044.2003 12819093 PMC161982

[52] Redpath SA, van der Werf N, Cervera AM, MacDonald AS, Gray D, et al. (2013) ICOS controls Foxp3(+) regulatory T-cell expansion, maintenance and IL-10 production during helminth infection. Eur J Immunol 43: 705–715. 10.1002/eji.201242794 23319295 PMC3615169

[53] Hammerich L, Heymann F, Tacke F (2011) Role of IL-17 and Th17 cells in liver diseases. Clin Dev Immunol 2011: 345803 10.1155/2011/345803 21197451 PMC3010664

[54] Barron L, Wynn TA (2011) Fibrosis is regulated by Th2 and Th17 responses and by dynamic interactions between fibroblasts and macrophages. Am J Physiol Gastrointest Liver Physiol 300: G723–G728. 10.1152/ajpgi.00414.2010 21292997 PMC3302189

[55] Zhang JY, Zhang Z, Lin F, Zou ZS, Xu RN (2010) Interleukin-17-producing CD4(+) T cells increase with severity of liver damage in patients with chronic hepatitis B. Hepatology 51: 81–91. 10.1002/hep.23273 19842207

