# Supplementary figures and images for: Th17 Down-regulation Is Involved in Reduced Progression of Schistosomiasis Fibrosis in ICOSL KO Mice
Source: PLoS Negl Trop Dis. 2015 Jan 15;9(1):e0003434. doi: 10.1371/journal.pntd.0003434 (PMC4295877; doi:10.1371/journal.pntd.0003434)

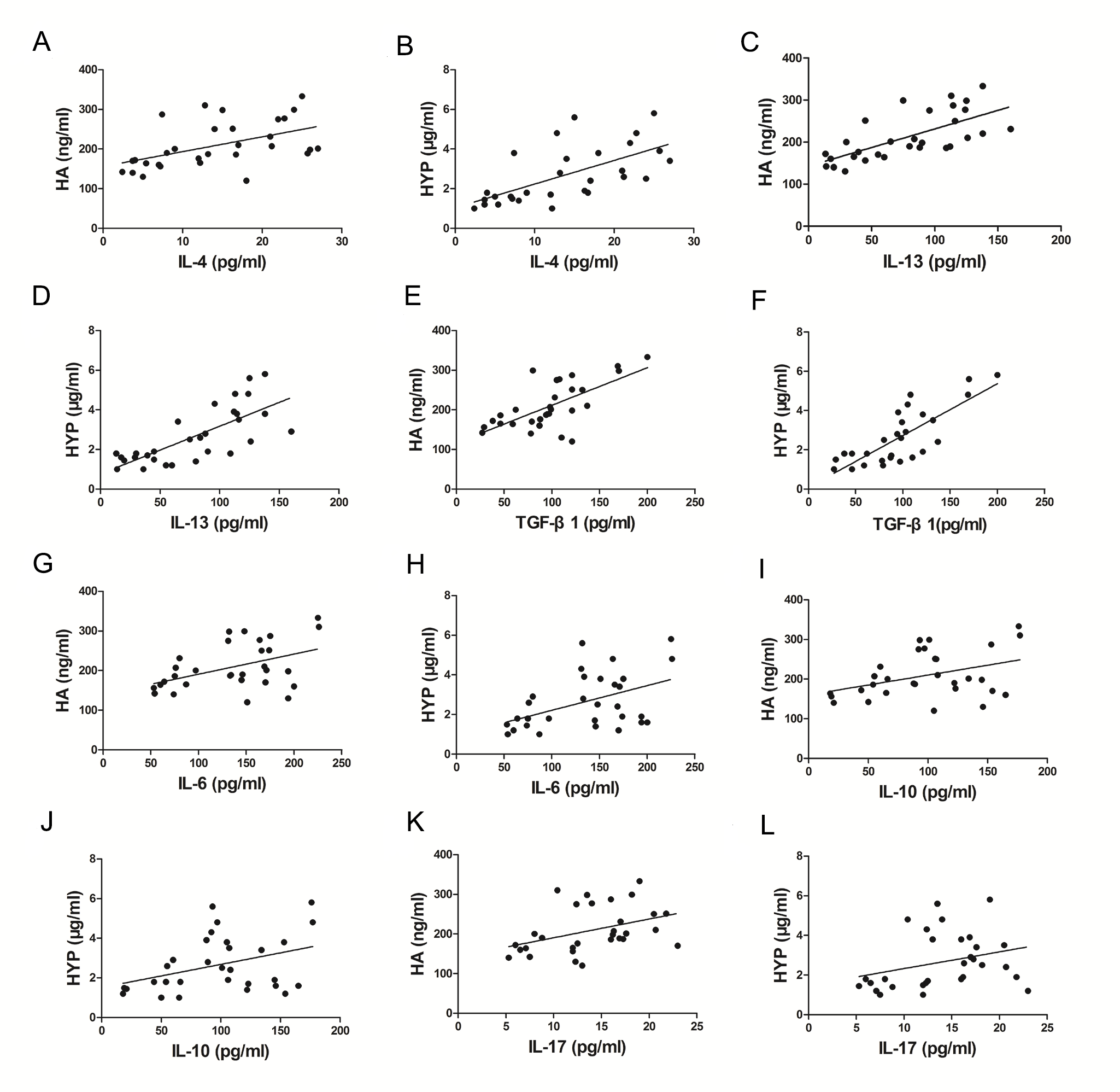

Supplement: S1 Fig — The linear relationship of IL-4 (A: R = 0.5858, ***P = 0.0007), IL-13 (C:R = 0.7432, ***P<0.0001), TGF-β1 (E:R = 0.6257, ***P = 0.0002), IL-10 (G:R = 0.3548, P = 0.0543), IL-17A (I:R = 0.4998, **P = 0.0049) and HA. The linear relationship of IL-4 (B:R = 0.7296, ***P<0.0001), IL-13 (D:R = 0.7732, ***P<0.0001), TGF-β1 (F:R = 0.7462, ***P<0.0001), IL-10 (H: R = 0.3171, P = 0.0877), IL-17A (J:R = 0.4241, *P = 0.0195) and HYP. The results are representative of three independent experiments with similar results, which arefrom fiveindependent mice in each group at 6 time points (0, 4, 7, 12, 16, 20 weeks). (TIF) [file pntd.0003434.s001.tif]

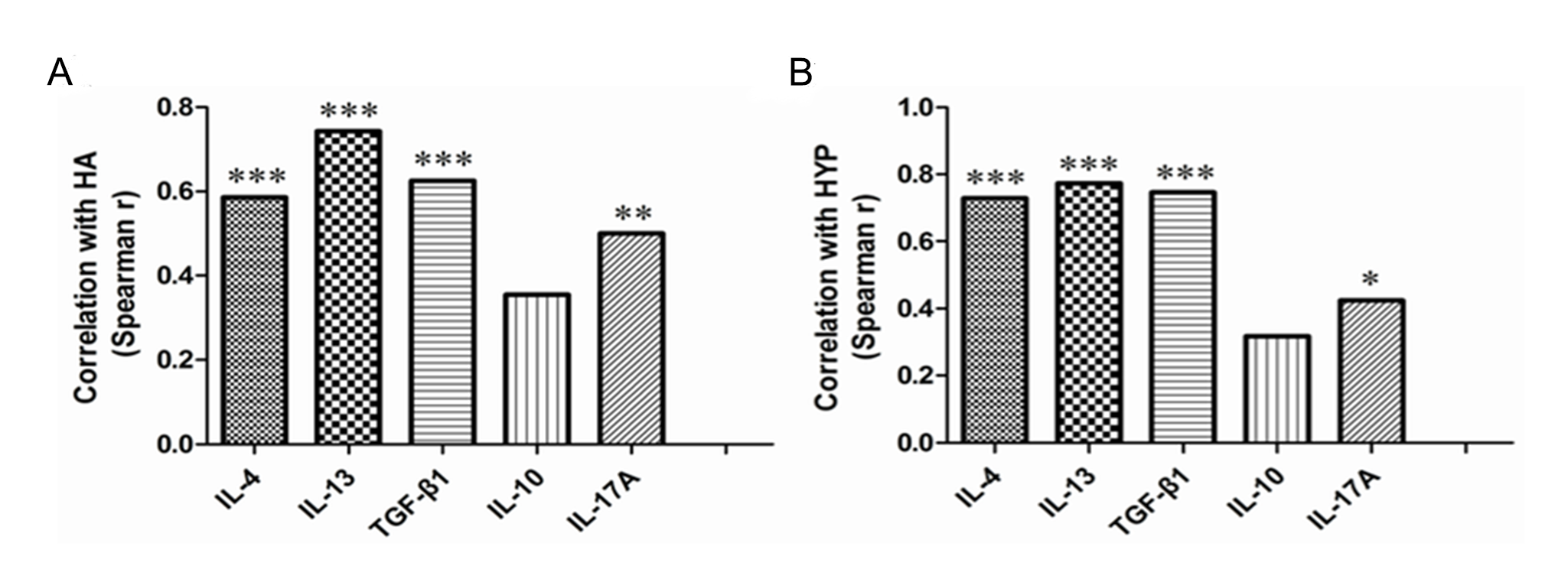

Supplement: S2 Fig — (A). The Spearman r from the analysis of correlation between IL-4/IL-13/TGF-β1/IL-10/IL-17A and HA. (B). The Spearman r from the analysis of correlation between IL-4/IL-13/TGF-β1/IL-10/IL-17A and HYP. *P<0.05, **P<0.01, and ***P<0.001 between cytokine and HA/HYP, Spearman’s rank. (TIF) [file pntd.0003434.s002.tif]

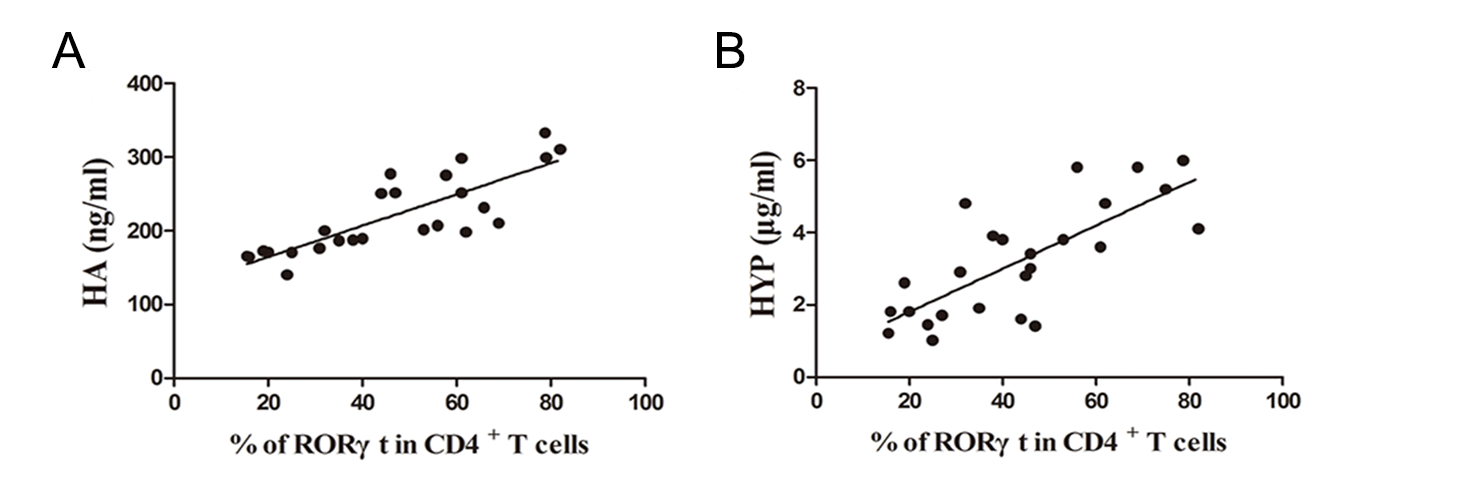

Supplement: S3 Fig — (A). The linear relationship of RORγt+ cells in CD4+ T cellsand HA (r = 0.8653, ***P<0.0001). (B). The linear relationship of RORγt+ cells in CD4+ T cellsand HYP (r = 0.7292, ***P<0.0001). The results are representative of three independent experiments with similar results, which are from five independent mice in each group at 5 time points (0, 4, 7, 12, 16 weeks). (TIF) [file pntd.0003434.s003.tif]
